# Strategies for hyperkalemia management in dialysis patients: A systematic review

**DOI:** 10.1515/med-2025-1301

**Published:** 2025-10-30

**Authors:** Anneliese Zevallos-Aquije, Axel Zevallos-Aquije, Rosa Alejandra Salas-Bolaños, Alvaro Maravi-Cardenas, Karen Palomino-Salcedo

**Affiliations:** Faculty of Human Medicine, Ricardo Palma University, Lima, Peru; Vice-Rectorate of Research, César Vallejo University, Trujillo, Peru; Department of Human Sciences, Universidad Internacional de La Rioja (UNIR), Logroño, Spain

**Keywords:** hyperkalemia management, dialysis treatment, chronic kidney disease, electrolyte imbalance, diuretic

## Abstract

**Background:**

Hyperkalemia is a potentially life-threatening electrolyte disorder, especially in patients with chronic kidney disease and those undergoing dialysis. Its management is complex due to the need to balance potassium control with overall patient stability.

**Objectives:**

The aim of this systematic review is to evaluate current therapeutic strategies for hyperkalemia in dialysis patients, including diuretics, ion-exchange resins, and newer agents such as sodium zirconium cyclosilicate (SZC).

**Methods:**

A systematic search was conducted in Scopus and Web of Science, applying filters for language, recency (≤5 years), and journal quality. After removing duplicates and irrelevant records, 11 high-quality studies were included.

**Results:**

New therapies like SZC and patiromer demonstrated efficacy in maintaining safe potassium levels. The potassium binding pack showed promise in acute and resource-limited settings. Evidence challenges strict dietary potassium restrictions, especially regarding plant-based foods, and highlights the importance of individualized nutritional plans. Continuous potassium monitoring is essential to preserve residual kidney function.

**Conclusion:**

Hyperkalemia management in dialysis patients benefits from an integrated approach combining pharmacologic treatment, tailored nutrition, and close monitoring. Novel interventions and evolving dietary guidelines may improve safety, effectiveness, and quality of life in this vulnerable population.

## 1 Introduction

The management of hyperkalemia, characterized by elevated potassium levels in the blood, is a significant clinical challenge, particularly in the treatment of patients with renal or cardiac diseases. Diuretics, especially loop diuretics and thiazide diuretics, play a crucial role in reducing potassium levels by increasing their renal excretion. However, their use must be carefully evaluated due to associated risks and the variability in patient response. Loop diuretics inhibit sodium and chloride reabsorption in the ascending limb of the loop of Henle, resulting in an increased excretion of potassium and other electrolytes [1]. However, their effectiveness may be compromised in patients with severe renal impairment (eGFR < 15 mL/min/1.73 m^2^) or those undergoing dialysis, where urine production is minimal [2]. Conversely, potassium-sparing diuretics, such as spironolactone, which act in the collecting duct to prevent potassium excretion, can exacerbate hyperkalemia rather than treat it, highlighting the need for an individualized treatment approach [3]. In the context of chronic kidney disease (CKD) and cardiac disease, diuretics may activate neurohormonal systems that affect glomerular hemodynamics, limiting their long-term efficacy [4]. This suggests that although diuretics are valuable tools in hyperkalemia management, their use should be part of a broader approach that includes evaluating therapy with renin-angiotensin-aldosterone system (RAAS) inhibitors and considering potassium-binding agents [5]. Therefore, while diuretics are fundamental in managing hyperkalemia, their use must be closely monitored and tailored to the individual needs of each patient. Preventing hyperkalemia episodes in dialysis patients is crucial, as this condition can lead to life-threatening complications. There are several highly effective strategies available, one of the most important being nutritional education, which enables patients to understand the necessity of avoiding excessive dietary potassium. Customizing dietary plans to account for individual preferences and needs has been shown to effectively reduce potassium levels [6,7]. In addition to dietary modifications, the use of ion-exchange resins is another effective strategy. These agents, which increase potassium elimination through the gastrointestinal tract, have been approved for the treatment of hyperkalemia since 1958 and remain a valid option in clinical practice [8]. Among other approved drugs for the treatment of hyperkalemia with a similar mechanism of action are patiromer and sodium zirconium cyclosilicate (SZC), both of which have demonstrated favorable outcomes in patients with CKD [9]. Furthermore, effective blood pressure management through antihypertensive medications and lifestyle changes can help prevent hyperkalemia episodes.

It is essential to review current hyperkalemia management strategies in dialysis patients, given its high prevalence and significant clinical implications. Both acute and chronic hyperkalemia can lead to severe complications, including cardiac arrhythmias and, in extreme cases, sudden death [10,11]. In dialysis patients, potassium regulation is critical, as these individuals have a reduced capacity to excrete potassium due to renal failure. Therefore, it is essential to implement effective strategies for its management and prevention [12]. Reviewing current strategies can help identify gaps in management and develop more effective interventions to improve the quality of life for dialysis patients [13,14].

## 2 Method

A systematic review was conducted to analyze current strategies that have proven effective in managing hyperkalemia, with the aim of identifying the most optimal interventions. Data collection utilized two main databases, Scopus and Web of Science, chosen for their rigorous indexing of academic publications and their inclusion of high-impact journals, which makes these databases prestigious sources. The following search commands were applied in both cases: in Scopus, (“potassium” OR “diuretics”) AND “dialysis” AND “hyperkalemia,” resulting in 1,418 publications; and in Web of Science, (“potassium” OR “diuretics”) AND “dialysis” AND “hyperkalemia,” yielding a total of 341 publications.

The review initially began with a total of 1,759 publications. During the screening process, strict exclusion criteria were applied to ensure the relevance and quality of the final content. First, 256 duplicate publications were removed. Next a language filter was applied, retaining only publications in English, which resulted in the exclusion of an additional 121 documents. Subsequently, 916 publications that were more than 5 years old were excluded, as the nature of this review required updated scientific findings; hence, the 5-year filter was applied. Finally, the document type was restricted to academic articles, leading to the removal of 219 publications.

As a result of these filters, 247 publications remained and were subjected to a detailed analysis of their abstracts, keywords, topics, and research areas. This process led to the exclusion of 235 additional publications. Ultimately, 12 publications were deemed relevant in terms of thematic focus, relevance, and objective results. From this group, applying a strict criterion based on the journal’s quality and its position in the highest quartile of the Scimago Journal Rank (SJR), 11 publications were selected to form the final corpus for this review.

**Figure 1:**
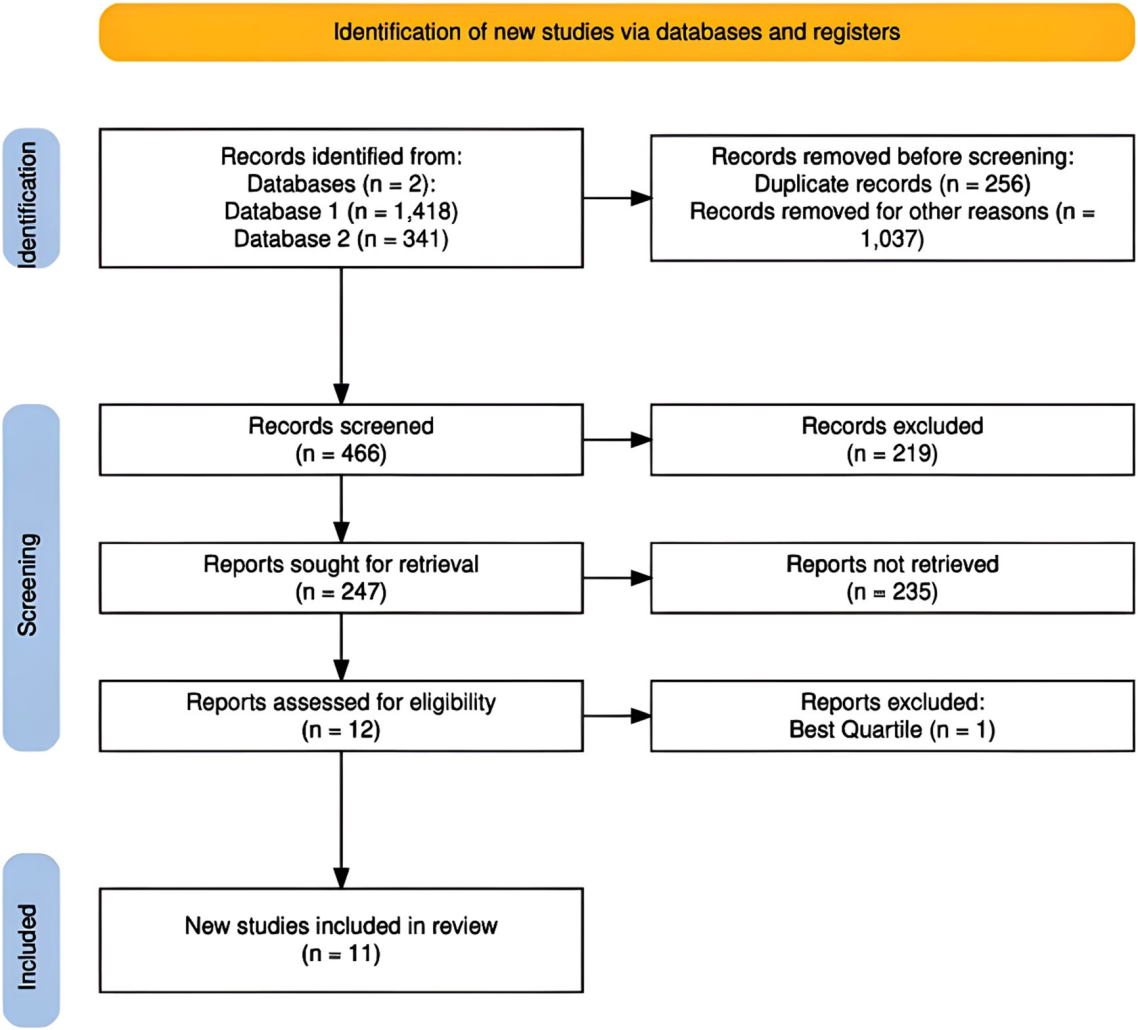
PRISMA flow diagram – study inclusion.

Figure 1 illustrates the flow diagram summarizing the study selection process [15]. From an initial search of 1,759 publications, exclusion filters were applied, reducing the number of studies to the 9 ultimately selected for this review. The diagram provides a clear visual representation of the systematic approach followed to ensure the quality and relevance of the included articles.

This systematic review was conducted in accordance with the PRISMA 2020 guidelines for reporting. Two independent reviewers screened the titles and abstracts, evaluated the full texts of eligible studies, and resolved any discrepancies through discussion. No automation tools were used during the selection process. Data were manually extracted into a structured Excel sheet, and articles were selected based on thematic relevance, study quality, and clinical applicability.

Table 1 summarizes 11 key studies on hyperkalemia, organized by authors, titles, sources, year, main disturbances, and performance. It includes relevant clinical cases, innovative treatments, and experimental studies, highlighting their clinical impact and quality based on the SJR.

**Table 1:** Summary of key studies on hyperkalemia management in dialysis patients

Authors	Titles	Source/Scimago Best Quartile	Year	Disturbance	Performance
Furusawa et al. [16]	A Case of TLS During Palliative Radiotherapy for Breast Cancer Metastases	Case Reports in Oncology/Q3	2023	TLS, a condition characterized by rapid tumor cell breakdown following cancer treatment. This leads to elevated levels of uric acid (hyperuricemia), potassium (hyperkalemia), and phosphorus (hyperphosphatemia), as well as decreased levels of calcium (hypocalcemia). These imbalances are critical and require prompt recognition and management to stabilize the patient’s condition.	TLS is a severe complication that can arise after cancer treatment, characterized by rapid tumor cell breakdown leading to elevated uric acid, potassium, and phosphorus levels, and decreased calcium. A case of a 65-year-old woman with advanced breast cancer is presented, who developed respiratory distress, tachycardia, and hypotension 11 h after palliative radiotherapy for cervical lymph node metastasis. Blood tests confirmed TLS with hyperuricemia and hyperkalemia. Prompt treatment with dialysis and electrolyte correction stabilized her condition. This case highlights the need for quick recognition and management of TLS in patients after radiation therapy
Song et al. [17]	A Case Report of Very Severe Hyperphosphatemia (19.3 mg/dL) in a Uremic Patient Taking Honey and Persimmon Vinegar	Electrolyte and Blood Pressure/Q3	2021	Hyperphosphatemia (elevated serum phosphate levels at 19.3 mg/dL) and hypocalcemia (low calcium levels). These imbalances were corrected through emergent HD, which led to significant improvement in the patient’s condition.	A 55-year-old male with type 2 diabetes, hypertension, and advanced CKD presented with weakness and altered mental status after refusing HD and self-medicating with honey and persimmon vinegar. Severe lab findings included blood urea nitrogen at 183.4 mg/dL and serum phosphate at 19.3 mg/dL, alongside a spontaneous subdural hemorrhage on CT. Emergent HD corrected his hyperphosphatemia and hypocalcemia, leading to significant improvement. This case emphasizes the importance of dietary restrictions and patient education for better adherence in CKD management.
Zimmerman et al. [18]	A Novel Peritoneal Packing Method for Management of Hyperkalemia During Acute Kidney Injury in Trauma	Military Medicine/Q3	2024	Hyperkalemia (high potassium), the study focuses on the use of the PBP to manage severe hyperkalemia, showing that the PBP group had significantly lower serum potassium levels compared to the control group, indicating its effectiveness in reducing hyperkalemia.	This study examines the PBP, a novel peritoneal packing material for managing severe hyperkalemia in resource-limited military settings. Male swine underwent nephrectomy and were divided into two groups: one receiving PBP and a control group with sham packs. After inducing hyperkalemia, potassium levels were measured over 12 h. Results indicated that the PBP group had significantly lower serum potassium levels compared to controls at 540 and 720 min (*P* = 0.006 and *P* = 0.015) and consistently lower dialysate potassium levels throughout the experiment (*P* < 0.001). These findings suggest that PBP effectively reduces hyperkalemia in trauma situations, highlighting its potential clinical application, though further validation is needed.
Fishbane et al. [19].	A phase 3B, randomized, double-blind, placebo-controlled study of SZC for reducing the incidence of predialysis hyperkalemia	Journal of the American Society of Nephrology/Q1	2019	Hyperkalemia, the study investigates the use of SZC in managing predialysis hyperkalemia in HD patients. The results showed that SZC effectively maintained potassium levels within the normal range in a higher percentage of patients compared to the placebo group.	The DIALIZE study (NCT03303521) tested SZC for predialysis hyperkalemia in HD patients. In this trial, 196 adults received either SZC (5–15 g daily) or placebo on non-dialysis days for 4 weeks. Results showed that 41.2% of SZC patients maintained potassium levels of 4.0–5.0 mmol/L, compared to 1.0% on placebo (*P* < 0.001). Additionally, rescue therapy for hyperkalemia was needed in 2.1% of SZC patients vs 5.1% in the placebo group. Serious adverse events were similar between groups. The study concluded that SZC is an effective and well-tolerated treatment for predialysis hyperkalemia in patients undergoing HD.
Kaarup et al. [20].	A question to the physician from a patient on HD: Would it be safe to run a marathon? A case report	HD International/Q3	2023	Severe hyperkalemia, which was not observed in the blood analyses before and after the endurance runs. The study suggests that endurance exercise, such as running, does not increase the risk of severe hyperkalemia in HD patients.	In a clinical study investigating the safety of endurance running for HD patients, an anuric patient completed eight runs, with a maximum distance of 32.2 km. Blood analyses conducted before and after these runs showed no instances of severe hyperkalemia. The findings suggest that there is no increased risk associated with endurance exercise, indicating that running may be safe for patients on maintenance HD
Cañas et al. [21].	A randomized study to compare oral potassium binders in the treatment of acute hyperkalemia	BMC Nephrology/Q2	2023	Hyperkalemia, the study focuses on the treatment of acute hyperkalemia in patients with potassium levels ≥ 5.5 mEq/L using various oral potassium binders.	The KBindER trial examines oral potassium binders for acute hyperkalemia in patients with potassium levels ≥ 5.5 mEq/L. Participants are randomized into four groups: sodium polystyrene sulfonate, patiromer, SZC, or a laxative. Primary endpoints include changes in potassium levels at 2 and 4 h, with additional analyses on hospital stay, next-morning potassium levels, and side effects. The study aims to enroll 80 patients to guide clinical use of oral potassium binders.
Takia et al. [22].	Acute Diarrhea and Severe Dehydration in Children: Does Non-anion Gap Component of Severe Metabolic Acidemia Need More Attention?	Indian Journal of Critical Care Medicine/Q2	2022	Hyperchloremia (elevated chloride levels) and hypernatremia (elevated sodium levels). Additionally, the study mentions acute kidney injury (AKI, a complication related to electrolyte imbalances and metabolic acidemia.	This study on children with acute diarrhea and severe non-anion-gap metabolic acidemia (sNAGMA) found that among 121 patients (median age 4 months), the median pH was 7.11, with 21% having pH < 7.00. Common complications included hyperchloremia (96%), hypernatremia (45%), and acute kidney injury (58%). The median time to resolve acidemia was 24 h, and adverse outcomes occurred in 32 patients. Higher grades of sNAGMA were linked to shock, AKI, and longer recovery times. The findings suggest a need for further trials on bicarbonate therapy in this population.
Yamaguchi et al. [23].	Association between annual variability of potassium levels and prognosis in patients undergoing HD	Clinical and Experimental Nephrology/Q2	2023	Hyperkalemia and hypokalemia. The study investigates the association between potassium level fluctuations and mortality in patients undergoing HD. It highlights that variability in serum potassium levels, rather than just the mean potassium level, is associated with prognosis and mortality in these patients.	The study demonstrated that variability in serum potassium levels is associated with mortality among HD patients, regardless of their average potassium levels. Among the 302 patients analyzed, 135 died during the 5-year observation period, and those with higher potassium variability had a significantly increased risk of mortality (adjusted hazard ratio: 6.93, 95% CI 1.98–25.00, *P* = 0.001). Patients in the highest tertile of variability (T3) had nearly twice the relative risk compared to those in the lowest tertile (T1). These findings highlight the importance of carefully monitoring potassium level fluctuations in this population to improve outcomes.
Arif et al. [24].	Association of serum potassium with decline in RKF in incident HD patients	Nephrology Dialysis Transplantation/Q1	2022	Hyperkalemia, the study discusses the relationship between serum potassium levels and the decline in RKF among HD patients, showing that higher potassium levels are associated with a greater decline in RKF.	The study found that hyperkalemia is associated with a decline in RKF among patients starting HD. Analyzing 6,655 patients, higher serum potassium levels correlated with greater reductions in renal urea clearance (KRU), with the steepest decline (−0.20, 95% CI −0.50 to −0.06) observed in patients with potassium levels > 5.0 mEq/L. Mediation analysis revealed that changes in KRU accounted for 1.78% of the relationship between serum potassium and mortality. These results highlight the impact of hyperkalemia on RKF and underscore the need for targeted management in advanced CKD, pending further clinical trial validation.
Babich et al. [25].	Taking the Kale out of Hyperkalemia: Plant Foods and Serum Potassium in Patients With Kidney Disease	Journal of renal nutrition/Q2	2022	Hyperkalemia in patients with CKD, challenging traditional dietary restrictions on potassium from plant-based foods. Recent studies suggest that dietary potassium does not always correlate with serum potassium levels due to factors like fiber, the alkalinizing effects of fruits and vegetables, and the bioavailability of potassium from plants. Additionally, plant-based foods provide benefits for kidney health, prompting recommendations to encourage their consumption rather than restrict them. The topic relates to potassium, CKD, and potential dialysis and hyperkalemia.	Recent studies challenge the traditional restriction of potassium in diets for kidney disease, particularly in patients with CKD. Evidence shows that dietary potassium, especially from plant-based foods, does not directly correlate with serum potassium levels due to factors such as the alkalinizing effects of fruits and vegetables, the bioavailability of plant potassium, and the impact of fiber on colonic potassium absorption. Additionally, plant-based foods may provide additional health benefits for CKD patients. Therefore, modern dietary guidelines should avoid unnecessary potassium restrictions from plant sources, encouraging patient-centered kidney-friendly recipes.
Vaz de Melo Ribeiro et al. [26].	Effect of a Nutritional Intervention, Based on Transtheoretical Model, on Metabolic Markers and Food Consumption of Individuals Undergoing HD	Journal of renal nutrition/Q2	2020	Notably, it reduced hyperkalemia and hyperphosphatemia, while increasing markers of iron metabolism and protein levels. This study highlights that a nutritional intervention based on the transtheoretical model significantly improved metabolic markers and dietary intake in individuals undergoing HD. Improvements were linked to better-calibrated caloric, macronutrient, and micronutrient intake, along with the use of phosphorus binders, demonstrating the essential role of nutrition in managing metabolic disturbances in HD patients.	The study found that the nutritional intervention based on the transtheoretical model led to significant behavioral changes in individuals undergoing HD, moving from the contemplation stage to the action stage (*P* < 0.001). There was a notable reduction in serum creatinine and pre- and post-dialysis urea levels (*P* < 0.001), as well as a significant decrease in hyperphosphatemia and hyperkalemia, along with improvements in bone metabolism markers (*P* < 0.001). Additionally, iron metabolism markers, protein, and globulin levels showed significant increases (*P* < 0.001 for iron, *P* = 0.042 for protein, and *P* < 0.001 for globulin). Dietary intake also improved significantly after the intervention, with increased caloric intake, cholesterol, protein, lipids, and micronutrients such as iron, phosphorus, potassium, copper, and vitamin C (*P* < 0.001). Overall, the intervention demonstrated the crucial role of nutrition in improving metabolic control among this patient group.

Note. This table summarizes and synthesizes data from selected peer-reviewed articles. Its purpose is to support the present literature review and contribute to the academic discussion while properly crediting the original authors.

## 3 Discussion

Through various studies and clinical cases, it has been demonstrated how electrolyte imbalances, such as altered potassium levels, can lead to severe complications, including cardiac arrhythmias, muscle dysfunction, and even death in extreme cases. This condition is particularly relevant in patients with kidney diseases, such as those undergoing hemodialysis (HD), as impaired renal function limits the body’s ability to properly excrete potassium. Several studies have explored electrolyte disorders and their clinical implications in different contexts.

One reported case describes tumor lysis syndrome (TLS), a severe complication characterized by rapid cellular destruction following oncological treatment. This syndrome was observed in a patient with advanced breast cancer who, after receiving palliative radiotherapy, developed metabolic disturbances such as hyperuricemia and hyperkalemia. These required urgent management with dialysis and electrolyte correction, stabilizing her condition [16]. Another case describes severe hyperphosphatemia in a man with advanced CKD, who experienced serious metabolic complications and subdural hemorrhage after self-medicating. Emergency HD successfully corrected the electrolyte imbalances, significantly improving his clinical status [17].

Experimental studies have also evaluated a novel material, referred to as the potassium binding pack (PBP), for managing hyperkalemia in traumatic contexts. The results showed a significant reduction in potassium levels, underscoring its potential clinical application in resource-limited settings, although further validation is required [18].

In the context of pre-dialysis hyperkalemia, one investigation tested the efficacy of SZC in HD patients. The study demonstrated that this compound effectively maintained potassium levels within normal ranges in a significant proportion of cases, with adverse events comparable to placebo [19]. Additionally, an analysis on the safety of physical exercise in HD patients concluded that resistance activities, such as marathon running, do not increase the risk of severe hyperkalemia under controlled conditions [20].

Another trial focused on comparing various oral potassium binders for the treatment of acute hyperkalemia, assessing their efficacy in reducing potassium levels within a few hours [21]. In children with severe acute diarrhea, complications such as hyperchloremia, hypernatremia, and acute kidney injury associated with severe metabolic acidosis were identified. This study highlights the importance of investigating new therapeutic strategies, such as bicarbonate supplementation [22].

Additional research explores the relationship between potassium variability and mortality in HD patients, as well as the impact of hyperkalemia on the loss of residual kidney function (RKF) in this group, emphasizing the importance of rigorous and individualized monitoring to improve clinical outcomes [23,24].

A comparative synthesis of the reviewed studies indicates that SZC offers consistent control of serum potassium with a favorable safety profile, often outperforming older agents like sodium polystyrene sulfonate in both efficacy and tolerability [19,21]. In contrast, ion-exchange resins, although widely used, present limitations in terms of delayed onset and gastrointestinal side effects. The PBP, evaluated in preclinical models, demonstrated rapid potassium reduction under extreme conditions, underscoring its potential utility in trauma or resource-limited environments [18]. These comparisons highlight the evolution of treatment strategies toward more personalized and effective hyperkalemia management.

Recent studies on CKD question the traditional dietary potassium restriction in these patients, particularly regarding plant-based foods. These foods may provide additional renal health benefits, suggesting they should be promoted rather than restricted, especially in the context of hyperkalemia and dialysis [25].

In addition, a nutritional intervention based on the transtheoretical model showed significant improvements in patients undergoing HD. It also enhanced the absorption of essential micronutrients such as potassium, phosphorus, and vitamin C. These findings underscore the importance of tailored nutritional approaches to manage metabolic disturbances in patients with renal failure [26].

However, dietary recommendations must be applied cautiously, particularly in patients with limited dialysis clearance or severe hyperkalemia. Although plant-based foods offer cardiovascular and metabolic advantages, their potassium content and absorption vary depending on preparation, fiber content, and individual tolerance [25]. In these cases, unrestricted intake may still increase the risk of potassium overload. Therefore, nutritional plans should be personalized and ideally guided by a renal dietitian to ensure both safety and adequacy.

## 4 Conclusion

Hyperkalemia is a severe electrolyte disturbance, particularly prevalent in patients with renal insufficiency and in complex clinical contexts such as TLS or the management of chronic diseases. Its association with higher mortality rates and renal function deterioration underscores the need for effective management strategies. Dialysis remains essential for correcting severe cases, while potassium binders, such as SZC, have proven effective for preventive control in HD patients.

Moreover, innovations like the PBP present promising alternatives in resource-limited settings, and continuous monitoring of potassium fluctuations is crucial due to their impact on prognosis. Recent research challenges the traditional dietary potassium restriction in CKD patients, especially when derived from plant-based foods. This is because such sources do not directly correlate with serum potassium levels due to factors such as fiber content and the bioavailability of plant-based potassium.

An integrated approach that combines acute therapies with preventive interventions and patient education is essential to stabilize affected individuals, prevent complications, and improve long-term clinical outcomes.
